# Higher synchronization stability with piano experience: relationship with finger and presentation modality

**DOI:** 10.1186/s40101-023-00327-2

**Published:** 2023-06-19

**Authors:** Kanami Ito, Tatsunori Watanabe, Takayuki Horinouchi, Takuya Matsumoto, Keisuke Yunoki, Haruki Ishida, Hikari Kirimoto

**Affiliations:** 1 Department of Sensorimotor Neuroscience, Graduate School of Biomedical and Health Sciences, Hiroshima University, 1-2-3 Kasumi, Minami-Ku, Hiroshima, 734-8553 Japan; 2 Faculty of Health Sciences, Aomori University of Health and Welfare, 58-1 Mase, Hamadate, Aomori, 030-8505 Japan; 3 Faculty of Health Sciences, Tokyo Kasei University, 2-15-1 Inariyama, Sayama, Saitama 350-1394 Japan

**Keywords:** Tapping, Sensorimotor synchronization, Multisensory integration, Finger motor control, Musical experience

## Abstract

**Background:**

Synchronous finger tapping to external sensory stimuli is more stable for audiovisual combined stimuli than sole auditory or visual stimuli. In addition, piano players are superior in synchronous tapping and manipulating the ring and little fingers as compared to inexperienced individuals. However, it is currently unknown whether the ability to synchronize to external sensory stimuli with the ring finger is at the level of the index finger in piano players. The aim of this study was to compare the effect of piano experience on synchronization stability between the index and ring fingers using auditory, visual, and audiovisual combined stimuli.

**Methods:**

Thirteen piano players and thirteen novices participated in this study. They were instructed to tap with their index or ring finger synchronously to auditory, visual, and audiovisual combined stimuli. The stimuli were presented from an electronic metronome at 1 Hz, and the tapping was performed 30 times in each condition. We analyzed standard deviation of intervals between the stimulus onset and the tap onset as synchronization stability.

**Results:**

Synchronization stability for visual stimuli was lower during ring than index finger tapping in novices; however, this decline was absent in piano players. Also, piano players showed the higher synchronization stability for audiovisual combined stimuli than sole visual and auditory stimuli when tapping with the index finger. On the other hand, in novices, synchronization stability was higher for audiovisual combined stimuli than only visual stimuli.

**Conclusions:**

These findings suggest that improvements of both sensorimotor processing and finger motor control by piano practice would contribute to superior synchronization stability.

## Background

Finger tapping tasks are widely used to study rhythmic motor control both with and without a pacing rhythm. In externally paced tapping, participants tap with their finger synchronously to external rhythms that are presented auditorily or visually, and the synchronization stability is commonly evaluated quantitatively using asynchrony time between the tap and stimulus presentation.

In previous studies, synchronization stability has been demonstrated to be higher for auditory than visual flash stimuli [1–4]. The possible explanation of this poor synchronization to visual flash stimuli is the longer temporal discrimination threshold for visual stimuli as compared with auditory stimuli [5] or the less frequent synchronized movements to visual stimuli in daily life [6]. Meanwhile, it has been reported that synchronization stability for visual stimuli can be improved to the level nearly close to that for auditory stimuli by adding their movement components. Specifically, synchronization is more accurate with a bouncing ball or an up-down moving bar (on a monitor) than visual flash stimuli [7–11]. Furthermore, recent studies have shown that synchronization stability is higher when tapping to audiovisual combined stimuli compared to sole auditory or visual stimuli [12–14].

In addition to the types of presentation modality (auditory, visual, and audiovisual), musical training can affect synchronization stability. Indeed, musically trained individuals can synchronize more accurately than novices with both auditory [15, 16] and visual [17] stimuli. Since musical experience has been shown to improve the ability of sensorimotor integration, as evidenced in shorter reaction times to auditory [18, 19], visual [20, 21], and audiovisual combined stimuli [22] compared to novices, the superior sensorimotor integration might be a key contributor to high synchronization ability in musicians. However, to our best knowledge, there are no studies that have compared the effect of piano experience on synchronization stability using different stimulus presentation modalities.

In regards to the musical experience, pianists who are expected to have extensive finger motor training have been reported to show remarkable finger tapping performance, and their piano training effects are particularly evident in the ring or little finger movements [23]. For example, although the maximum speed of two-finger tapping was slower with ring and little fingers than with index and middle fingers regardless of piano experience, a decline in performance from two-finger tapping with index and middle fingers to that with ring and little fingers was smaller in pianists than novices [23]. Even though this prior work has revealed that piano training can improve tapping performance particularly for the ring and little fingers, it is currently unknown whether the ability to synchronize to external sensory stimuli with the ring finger is at or similar to the level of the index finger in piano players. In this study, we focused on the ring finger because movement restrictions are greater in the ring than little finger given that the ring finger, unlike the little finger, lacks the muscle responsible solely for extension and thus is strongly influenced by the intertendinous connections between the tendons of the middle and little fingers [24].

Accordingly, the purpose of this study was to compare the effect of piano experience on synchronization stability between the index and ring fingers using different stimulus presentation modalities. To this end, piano players and novices tapped with their index or ring finger synchronously to auditory, visual, and audiovisual combined stimuli. In this study, we employed an electronic metronome (with moving visual stimuli) commonly used by musicians during practice as it could emphasize the effect of piano experience. We hypothesized that synchronization stability would be higher in piano players than novices particularly for ring finger tapping in all but especially in the audiovisual combined stimulus condition, and that synchronization stability would be higher for audiovisual combined stimuli than sole visual or auditory stimuli during both index and ring finger tappings in piano players. In terms of the perspective of physiological anthropology, elucidating the development of fine finger motor skills through piano practice could clarify how individuals adapt to musical cultures and provide essential data that advance research of human physiological function.

## Materials and methods

### Participants

Thirteen amateur piano players (4 males and 9 females, mean age ± SD = 22.8 ± 2.5) and thirteen novices (8 males and 5 females, mean age ± SD = 23.4 ± 3.5) participated in this study. The mean years of experience of piano players were 10.3 ± 4.3 years (range: 6–20 years). Novices have never received piano training. All the participants had normal or corrected-to-normal vision and were all right handed as confirmed by Edinburgh Handedness Inventory (all ≧ 80) [25]. Written informed consent was obtained from each participant prior to the start of the experiment. This study was approved by the Ethics Committee of Hiroshima University (E-2549) and conducted in accordance with the principles of the Declaration of Helsinki.

### Design and procedure

The participants sat on a chair with their forearms pronated and resting on the armrests and faced to an electronic metronome (TM-50, KOLG, Japan) which was set 30 cm in front of them at the height of the eye. The right forearm and wrist were fixed to the armrest. They performed an externally paced tapping task with their right index or ring finger by lightly hitting a force transducer (Tech Gihan, Kyoto, Japan) on a table that was set in front of the right armrest. The force transducer was placed just under the finger used for tapping. The force signal was low-pass filtered at 5 Hz. Signals from the force transducer and the metronome were digitized using an analog-to-digital converter (PowerLab, ADInstruments, Australia), sampled at 2 kHz, and stored on a personal computer for off-line analysis (LabChart 8.1.13, ADInstruments, Australia).

There were three conditions as follows: auditory, visual, and audiovisual combined conditions where the auditory and visual stimuli were presented simultaneously. In the auditory condition, participants were asked to synchronize their taps with the tone (1 kHz) presented by the electronic metronome through a headphone (G PRO X, Logicool, Japan). In the visual condition, they looked at a screen of the electronic metronome and were instructed to tap when a line which moving like a pendulum at a constant speed reached one of the edges of the screen. In the audiovisual combined condition, both the auditory and visual stimuli were presented simultaneously. Based on previous studies [4, 9, 26, 27], all the stimuli were presented at 1 Hz, and the participants performed 30 taps in each condition. They stayed still without tapping for the first 5 stimuli and started tapping from 6th stimulus. In the visual and audiovisual combined conditions, the participants were instructed to visually focus on the pendulum motion on the liquid crystal screen of the electronic metronome, and in the auditory condition, they were instructed to focus on the screen. Additionally, they were instructed to keep their wrist, palm, and the non-tapping fingers in contact with the armrest/tabletop and to maintain a constant tapping force during the task. No instructions were given regarding the tapping height. Before the experiment, the participants practiced the tapping task once for the audiovisual condition using each finger. Each participant completed six tapping tasks (3 conditions × 2 fingers). The order of the conditions was pseudo randomized among the participants.

### Data analysis

The data were analyzed with MATLAB R2021a (MathWorks, USA). The force signal was low-pass filtered at 10 Hz (fourth-order Butterworth filter) to remove noises and down-sampled to 1 kHz. Asynchrony time was calculated as a difference between tap onset and metronome onset. The tap onset was defined as the first time point at which the force signal reached above mean + 5 standard deviation (SD) of the baseline (− 400 to − 200 ms before each stimulus onset). We calculated SD of asynchrony time to evaluate synchronization stability.

### Statistical analysis

Statistical analysis was performed using SPSS Statistics software version 21 (SPSS, IBM Corp., USA). A three-way repeated measures analysis of variance (ANOVA) was used to examine the effects of musical experience, presentation modality, and finger on the asynchrony time and synchronization stability. Post hoc test was conducted with Bonferroni adjustment. In addition, Pearson’s correlation coefficients were calculated between the years of musical experience and synchronization stability in each condition for piano players. Significant level was set at *p* < 0.05.

## Results

For the mean asynchrony time, a three-way repeated measures ANOVA showed no significant main effect or interaction.

**Fig. 1:**
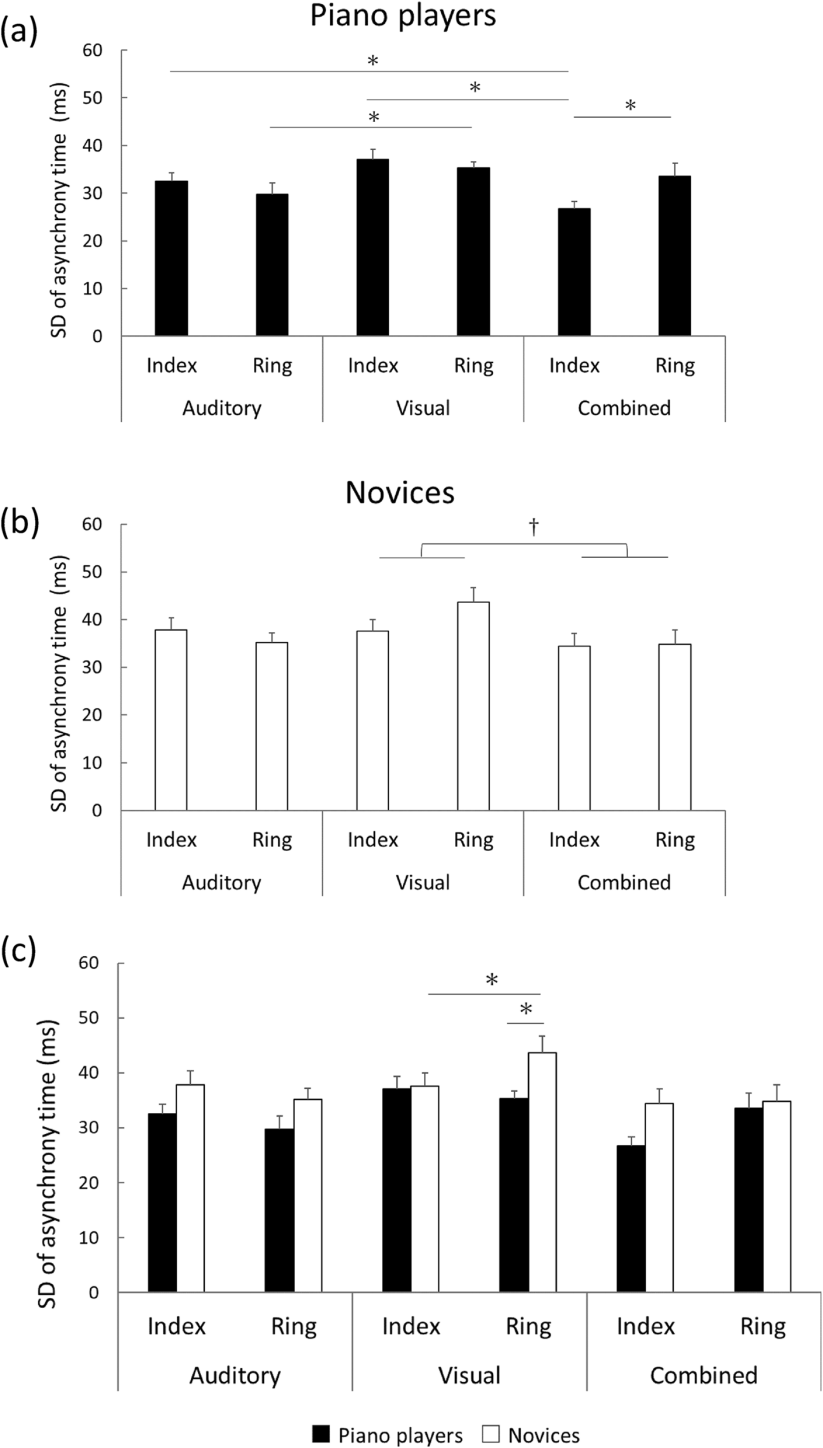
The SD of asynchrony time. **a** SD of asynchrony time in piano players: synchronization stability for the index finger was higher for audiovisual combined stimuli than sole visual or auditory stimuli. **b** SD of asynchrony time in novices: synchronization stability was higher for audiovisual combined stimuli than sole visual stimuli. **c** SD of asynchrony time in each stimulus modality: in the visual condition, synchronization stability for the ring finger was higher in piano players than novices, and also, the synchronization stability was lower during ring than index finger tapping only in novices. Error bars present standard error of the mean. The asterisks indicate significant post hoc differences

For the SDs of asynchrony time, a three-way repeated measures ANOVA revealed a main effect of Modality (*F*_(2,50)_ = 12.209, *p* < 0.001, partial *η*^2^ = 0.337), an interaction of Modality and Finger (*F*_(2,50)_ = 3.480, *p* = 0.039, partial *η*^2^ = 0.127), and an interaction of Group, Modality, and Finger (*F*_(2,50)_ = 3.972, *p* = 0.025, partial *η*^2^ = 0.142). Subsequent two-way ANOVA with factors Modality and Finger for the piano players revealed a main effect of Modality (*F*_(2,50)_ = 6.284, *p* = 0.006, partial *η*^2^ = 0.344) and an interaction of Modality and Finger (*F*_(2,50)_ = 6.672, *p* = 0.005, partial *η*^2^ = 0.357) (Fig. 1a). Post hoc analysis revealed that the SD of asynchrony time with index finger was smaller in the combined condition as compared to the auditory (*p* = 0.01) and visual (*p* < 0.001) conditions. Also, the SD of asynchrony time with ring finger was smaller in the auditory than visual condition (*p* = 0.029). Furthermore, in the combined condition, the SD of asynchrony time was smaller for index than ring finger (*p* = 0.017). On the other hand, subsequent two-way ANOVA with factors Modality and Finger for novices revealed a main effect of Modality (*F*_(2,50)_ = 5.983, *p* = 0.008, partial *η*^2^ = 0.333) (Fig. 1b). Post hoc analysis revealed that the SD of asynchrony time was smaller in the combined than visual condition (*p* = 0.003). In addition, we conducted subsequent two-way ANOVA with factors Group and Finger for the auditory, visual, and combined conditions (Fig. 1c). In the visual condition, there was a significant interaction of Group and Finger (*F*_(2,50)_ = 9.467, *p* = 0.005, partial *η*^2^ = 0.283). Post hoc analysis revealed that the SD of asynchrony time was larger with ring than index finger in novices (*p* = 0.003), and that the SD of asynchrony time with ring finger was larger in novices than piano players (*p* = 0.016). In the auditory or combined condition, there was no significant main effect or interaction of Group and Finger. Finally, we conducted subsequent two-way ANOVA with factors Group and Modality for the index and ring fingers. For the index finger, there was a significant main effect of Modality (*F*_(2,50)_ = 8.181, *p* = 0.001, partial *η*^2^ = 0.254), and post hoc analysis showed that the SD of asynchrony time was smaller in the combined than visual condition (*p* < 0.001). For the ring finger, there was a significant main effect of Modality (*F*_(2,50)_ = 7.824, *p* = 0.001, partial *η*^2^ = 0.246), and post hoc analysis showed that the SD of asynchrony time was smaller in the auditory and combined conditions as compared to the visual condition (auditory: *p* = 0.001, combined: *p* = 0.023).

Regarding the correlation between the years of musical experience and the SD of asynchrony time, no significant results were obtained in all the conditions.

## Discussion

In this study, we compared the effect of piano experience on synchronization stability between the index and ring fingers using different stimulus presentation modalities. As a result, synchronization stability during ring finger tapping was greater in piano players than novices in the visual condition. Moreover, we found that synchronization stability for visual stimuli was lower during ring finger tapping as compared to index finger tapping in novices; however, this decline in synchronization stability for visual stimuli during ring finger tapping was not apparent in piano players. In addition, synchronization stability during index finger tapping was greater for audiovisual combined stimuli as compared to sole visual and auditory stimuli in piano players. On the other hand, in novices, synchronization stability was greater for combined stimuli than only visual stimuli.

We found the effect of piano experience on synchronization stability in the visual condition with the ring finger. This finding indicates that synchronization stability for moving visual stimuli is affected by musical experience, particularly when using the finger with less dexterity. When both auditory and visual stimuli are presented with a phase shift, tapping rhythm is entrained to auditory stimuli [6]. This auditory dominance in synchronization tasks is probably due to high temporal resolution in auditory processing [5] and strong functional coupling between auditory and motor cortices [28]. On the other hand, the other studies have reported that synchronization to visual moving stimuli can be as accurate as auditory stimuli [7–10]. It has been demonstrated that synchronization stability to moving visual stimuli improves remarkably after the age of 10 years [29]. In addition, deaf individuals have been found to synchronize to visual stimuli more accurately than normal-hearing individuals [30]. These results suggest that synchronization stability for moving visual stimuli would improve through individual experience. Therefore, our finding of high synchronization stability for moving visual stimuli in piano players can be ascribed to rich experience of observing movements of a metronome. However, it should be noted that synchronization stability was higher for auditory and combined stimuli than visual stimuli regardless of tapping fingers in piano players. Because metronome rhythm is usually presented both auditorily and visually, synchronizing only to visual stimuli from the metronome might have been difficult for piano players.

Besides the stimulus presentation modality, the effect of piano experience on synchronization stability was pronounced in the ring finger. The independent movement of ring finger is limited as compared to the other fingers [31, 32], and this limitation is caused by differences in anatomical structure and neuromuscular control between fingers [24]. The movement of ring finger is thought to be affected by the intertendinous connections between the tendons of extensor digitorum muscles because there are no muscles that solely work for extension of the ring finger. On the other hand, pianists could move their ring finger faster and more accurately than novices [23], and this enhanced performance would be ascribed to plastic changes of neuromuscular control by piano practice [33]. Similarly, in string players who often use their little finger during the playing, the cortical representation of little finger responded more greatly to tactile input as compared to novices. Moreover, the distance between cortical representation of little finger and that of thumb was greater in string players as compared to novices [34]. These findings suggest that cortical activity could change after the use of specific fingers. Therefore, in piano players of the current study, neuromuscular control of ring finger and associated cortical activity may have changed after piano practice, which possibly resulted in a stable movement of ring finger.

We found that synchronization stability was improved by the use of audiovisual combined stimuli especially in piano players, particularly when using the index finger. In externally paced tapping, timing estimation based on external rhythms and sensorimotor integration is important. Previous studies have demonstrated that the measurement of time period between two stimuli was more accurate for audiovisual combined stimuli than sole auditory or visual stimuli [35], and that reaction times to audiovisual stimuli were faster than sole auditory or visual stimuli [22]. These findings indicate that time estimation and reactions to external stimuli can be enhanced by presentation of multisensory information. Moreover, responses of superior colliculus neurons, which play an important role in responding to multisensory information [36], have been found to be immature in newborn as compared to adult monkeys [37], suggesting that multisensory integration develops after rich experience with multisensory signals. Indeed, previous studies have shown that musical training enhances information processing and motor control associated with integration of multisensory information. For example, piano training induces plastic reorganizational changes in the auditory cortex, surpassing the effect of auditory training alone [38]. Also, a functional magnetic resonance imaging study has demonstrated that broader brain areas were recruited when listening and seeing a musical performance in piano players than novices, and the recruitment was observed especially in brain regions known to be related to integration of sensory and motor information [39]. Furthermore, the plastic brain changes related to the function of multisensory integration in piano players contribute to the neural basis of their advanced finger motor control [23, 40–43]. As precisely synchronizing tapping with both visual and auditory stimuli requires these functions, the high tapping synchronization stability for audiovisual stimuli in piano players, as demonstrated in our study, is believed to be due to the development of sensorimotor processing and fine motor control through piano experience. The reason why novices presented higher synchronization stability for audiovisual combined stimuli than only visual stimuli might be because of the auditory dominance mentioned above but needs to be studied further in future studies.

There are several limitations in this study. First, we recruited participants based only on their piano experience duration, and their performance frequency was different. Previous studies reported correlations between early onset of piano playing and high synchronization stability [44] and between total time of musical practice and surround inhibition of fingers [45]. Therefore, future studies on synchronization stability should be conducted with consideration of musical experience from various perspectives. Second, superiority of tapping performance of pianists might have been more apparent with different experimental settings. It has been reported that cortical response to somatosensory stimulation to the left little finger was larger for string players than controls [34]. Considering the impact that many piano players have experience in playing string instruments and can use their nondominant hand (mostly left hand) as proficiently as their dominant hand (mostly right hand) during piano performance, the use of left little finger (instead of the right ring finger) may have emphasized the superiority of tapping performance of piano players over novices. Furthermore, we adopted the stimulus presentation frequency of 1 Hz in this study. Given that pianists can play much faster than this frequency, the higher presentation frequency (and thus tapping frequency) might have amplified the differences in synchronization stability between piano players and novices. Third, we did not evaluate any neurophysiological data. Additional studies are warranted to reveal the neurophysiological difference between piano players and novices during externally paced tapping. Finally, this study focused on piano experience and finger motor control; therefore, it is uncertain whether our findings are limited to these contexts (i.e., instruments and body parts). Nonetheless, future comprehensive investigations with consideration with these factors will lead to progress in the field of physiological anthropology.

## Conclusion

The present study demonstrated that synchronization stability during ring finger tapping was higher in piano players than novices for visual moving stimuli. Also, synchronization stability was higher when tapping with audiovisual combined stimuli as compared to sole visual and auditory stimuli, especially in piano players. These findings suggest that improvements of both sensorimotor processing and finger motor control by piano practice would contribute to superior synchronization stability.

## Data Availability

The datasets used and/or analyzed during the current study are available from the corresponding author on reasonable request.

## Abbreviation

ANOVA: Analysis of variance
